# A long-term cohort study: the immune evasion and decreasing neutralization dominated the SARS-CoV-2 breakthrough infection

**DOI:** 10.3389/fcimb.2024.1381877

**Published:** 2024-03-20

**Authors:** Qianyun Liu, Meihua Jin, Fanghua Mei, Hui Fan, Mengxue Gu, Yuzhen Zhang, Shengnan Qian, Xue Tan, Lei Ji, Zhen Zhang, Guozhong Chen, Huan Yan, Yu Chen, Ke Lan, Qing Geng, Kun Cai, Li Zhou

**Affiliations:** ^1^ State Key Laboratory of Virology, Department of Thoracic Surgery, Renmin Hospital of Wuhan University, Wuhan, China; ^2^ State Key Laboratory of Virology, College of Life Sciences, TaiKang Center for Life and Medical Sciences, Wuhan University, Wuhan, China; ^3^ State Key Laboratory of Virology, Modern Virology Research Center and RNA Institute, College of Life Sciences and Frontier Science Center for Immunology and Metabolism, Wuhan University, Wuhan, China; ^4^ Huzhou Center for Disease Control and Prevention, Huzhou, China; ^5^ Hubei Center for Disease Control and Prevention, Wuhan, China; ^6^ Department of Respiratory and Critical Care Medicine, Renmin Hospital of Wuhan University, Wuhan, China; ^7^ Animal Biosafety Level (ABSL)-III Laboratory/Institute for Vaccine Research, Wuhan University, Wuhan, China

**Keywords:** SARS-CoV-2, omicron subvariants, immune evasion, immune imprint, long-tracked cohort, JN.1

## Abstract

Most of vaccinees and COVID-19 convalescents can build effective anti-SARS-CoV-2 humoral immunity, which helps preventing infection and alleviating symptoms. However, breakthrough viral infections caused by emerging SARS-CoV-2 variants, especially Omicron subvariants, still pose a serious threat to global health. By monitoring the viral infections and the sera neutralization ability of a long-tracked cohort, we found out that the immune evasion of emerging Omicron subvariants and the decreasing neutralization led to the mini-wave of SARS-CoV-2 breakthrough infections. Meanwhile, no significant difference had been found in the infectivity of tested SARS-CoV-2 variants, even though the affinity between human angiotensin-converting enzyme 2 (hACE2) and receptor-binding domain (RBDs) of tested variants showed an increasing trend. Notably, the immune imprinting of inactivated COVID-19 vaccine can be relieved by infections of BA.5.2 and XBB.1.5 variants sequentially. Our data reveal the rising reinfection risk of immune evasion variants like Omicron JN.1 in China, suggesting the importance of booster with updated vaccines.

## Introduction

The coronavirus disease 2019 (COVID-19) pandemic, caused by SARS-CoV-2, still rages around the world since December 2019 (Chen LJ. et al., 2020; Chen Y. et al., 2020; Zhou et al., 2020). Since the initial outbreak, SARS-CoV-2 has been constantly mutating, and most of mutations gather in the genes of structural proteins, especially the Spike, which interacts with human angiotensin-converting enzyme 2 (hACE2) for viral entry (Zhou et al., 2020; Hoffmann et al., 2020; Chen et al., 2022).

The Spike protein consists of the S1 and S2 subunits, and the receptor-binding domain (RBD) in the S1 subunit is responsible for hACE2 recognition. The mutations in the Spike, especially in the RBD, may affect the affinity between RBD and hACE2. For instance, mutation N501Y, initially identified in Alpha variants (also known as B.1.1.7), imparts cross-species transmission of SARS-CoV-2 to mice by enhancing receptor binding (Niu et al., 2021; Liu Y. et al., 2022). Meanwhile, mutations in the RBD are responsible for the immune evasion, because RBD is the major neutralizing antibody (nAb) epitope and RBD-specific nAbs are vital for serum neutralizing in vaccinees and COVID-19 convalescents. Mutations such as S371L, K417N, L452R, and E484K largely impact the immune response of vaccinees and convalescents by avoiding the antibody recognition of RBD while have little impact on the affinity between RBD and hACE2 (Chen et al., 2022; Liu Q. et al., 2022; Liu et al., 2023; Li et al., 2020; Harvey et al., 2021).

In the early stages of the COVID-19 pandemic, only several mutations in Spike were identified in Alpha, Beta, Gamma, and Delta variants. The Delta variant was once the dominant lineage, but since January 2022, it was replaced by the Omicron variant, which carries more than 30 mutations in the Spike protein. Although most of convalescents can built humoral immune against SARS-CoV-2 after infection, Omicron subvariants, such as BA.1–BA.4, BA.5.2, BQ.1.1, XBB, EG.5 and JN.1, are still emerging, causing mini-waves of breakthrough infection (infection or reinfection in vaccinees or convalescents) around the world. Investigation on the duration and broad neutralization of the humoral immune in COVID-19 convalescents can provide insights into the antibody immunity and protection against SARS-CoV-2 infection/reinfection and in developing broad and potent vaccines against the emerging immune evasive variants.

In this study, we tracked a 2-year-long cohort who had received three doses of inactivated COVID-19 vaccine based on the original strain in Wuhan, China. The sera samples were collected before and after the infection of Omicron BA.5.2 and the reinfection of Omicron XBB.1.5. By assessing the neutralizing activity of these sera, we found out that the constant mini-waves of SARS-CoV-2 infections caused by the emerging variants were related with the drastic immune evasion and the decreasing sera neutralization of convalescents along time. Meanwhile, repeated infections by Omicron subvariants largely mitigated the immune imprinting of inactivated vaccines. Overall, our results reveal the rising reinfection risk of emerging immune evasion variants and suggesting the importance of booster and the antigen update of vaccines.

## Results

### A long-tracked vaccinees repeatedly infected by Omicron variants

**Figure 1:**
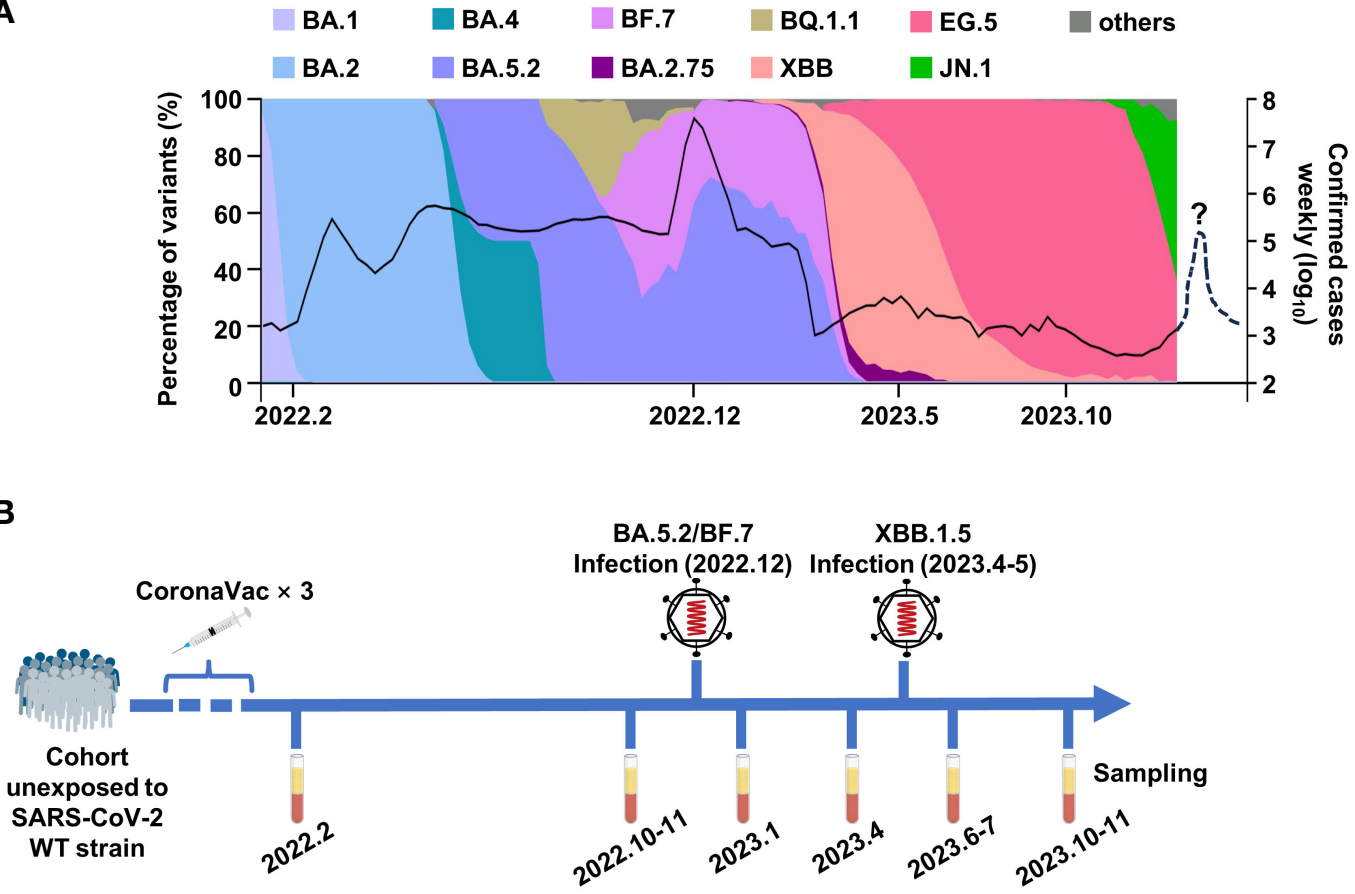
The breakthrough infections of BA.5.2/BF.7 and XBB.1.5 in China. **(A)** The composition ratio of SAS-CoV-2 variants and the confirmed COVID-19 cases in China. The data are from Chinese Center for Disease Control and Prevention and GISAID. The dashed line donates the possible mini-wave of SARS-CoV-2 infection in the future. **(B)** The timetable of sample collection for this study.

At the beginning of 2020, the COVID-19 caused a short chaos in China, but the epidemic was quickly brought under control, due to the strict and effective epidemic prevention and control policies. Along with the increasing vaccination rate, the control policies were gradually loosened. At the beginning of 2022, over 87% of people had been fully vaccinated, and most of the them were administrated with three doses of inactivated vaccine, which was an inactivated vaccine based on the original SARS-CoV-2 strain (www.gov.cn). The herd immunity built by inactivated vaccines had played important roles against breakthrough infection in China. However, over 80% of Chinese were infected by Omicron BA.5.2 or BF.7 variants from December 2022 to February 2023, leading to a peak of infection (**Figure 1A**). Fortunately, the case fatality rate of Omicron variants was significantly lower than the former strains, which could be explained by the vaccination, better medical conditions, and the decrease in the pathogenicity of the virus itself (Yuan and Ye, 2022; Bálint et al., 2022; Fan et al., 2022). After this infection peak, only mini-waves emerged, with the rapid changes in the circulating Omicron sublineages in China. It was worth noting that the Omicron XBB.1.5 caused a small infection peak in the summer and autumn of 2023 (**Figure 1A**).

To monitor the immune response in vaccinees, we tracked a cohort consisting 30 participants in Wuhan, who were infected by Omicron BA.5.2 subvariant in December 2022 and were infected by Omicron XBB.1.5 in April–May 2023. As shown in **Figure 1B**, the sera samples of this cohort were collected at multiple time points: 1 month and 9 months after the last dose inactivated vaccine, 1 month and 4 months after each infection.

### The infectivity of the Omicron subvariants

**Figure 2:**
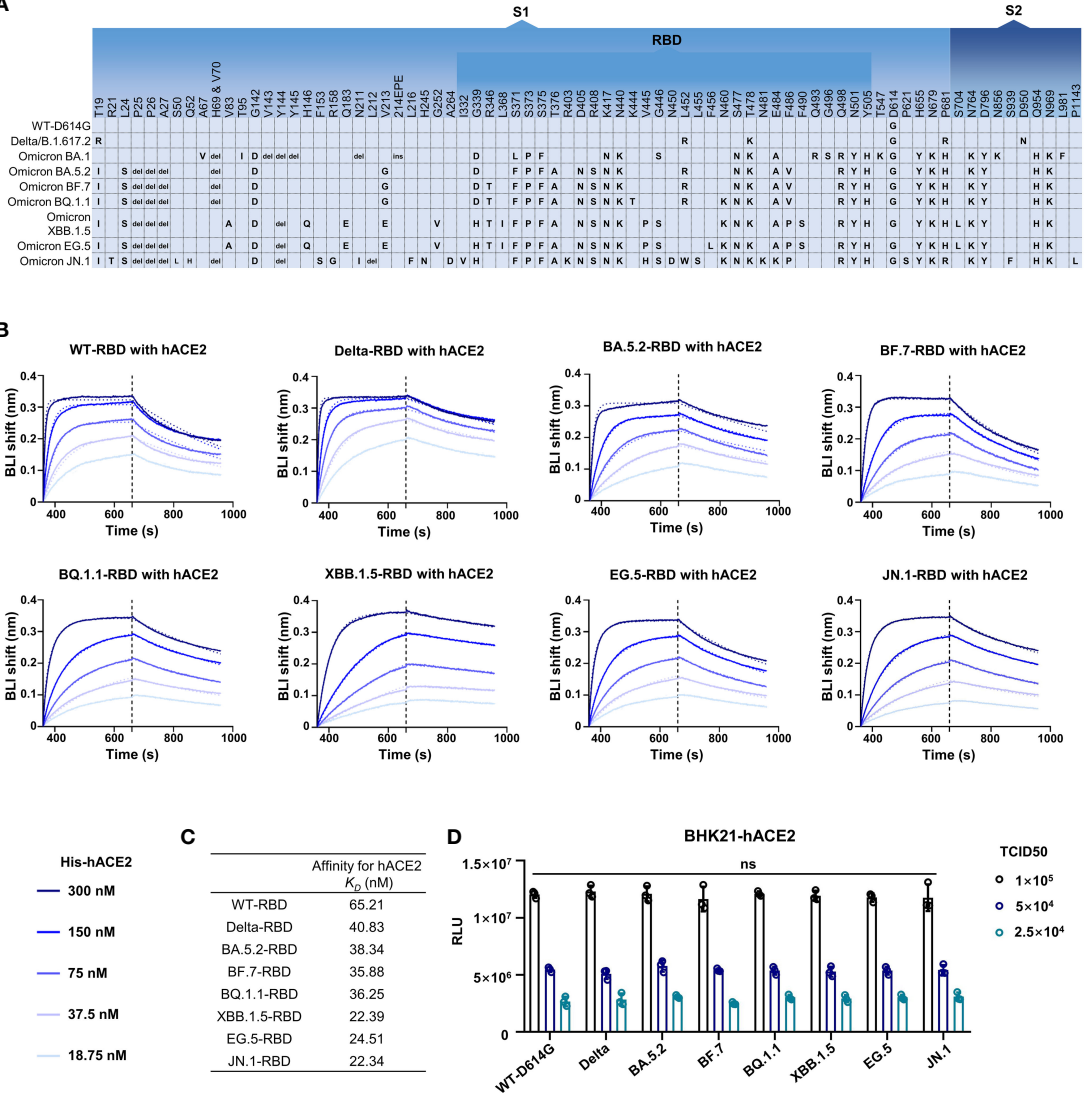
The infectivity of the SARS-CoV-2 and its variants. **(A)** The amino acid alterations in the S protein of SARS-CoV-2 variants tested in this study. **(B, C)** The hACE2 affinity test. Dashed lines are the fit lines. **(D)** The pseudovirus infection ability of SARS-CoV-2 variants as indicated. Two-way ANOVA with Tukey’s multiple comparison test was used. ns, *p* > 0.05. Error bars represent the means with standard deviations. Three times of replicates were tested and the average values were taken.

It was reported that D614G can significantly improve infectivity of SARS-CoV-2 (Zhou et al., 2021; Ma et al., 2022). The Omicron subvariants carried more than 30 mutations in addition to D614G in Spike (**Figure 2A**). In order to investigate the potential effect of mutations on the infectivity, we detected the hACE2 affinity and infection features of SARS-CoV-2 wild type (WT), Delta variant, and several Omicron subvariants. Through biolayer interferometry (BLI) assay, the hACE2 binding capacity of RBDs from SARS-CoV-2 WT and other seven previous/currently circulating variants were measured. The kinetic binding affinity (*K_D_*) values between RBDs and hACE2 were from 22.34  nmol/L to 65.21 nmol/L, showing an increasing trend of hACE2 affinity from WT to XBB.1.5 and JN.1 but still in same order magnitude (**Figures 2B, C**).

We then tested the infection features of these strains through pseudovirus infection assay. Pseudovirus bearing WT-Spike protein with D614G mutation (WT-D614G) was used as a reference virus as the D614G is critical for higher viral infectivity. Interestingly, no significant difference was observed in all tested strains including WT-D614G, Delta, Omicron BA.5.2, BF.7, BQ.1.1, XBB.1.5, EG.5, and JN.1 at the same median tissue culture infectious dose (TCID_50_) in BHK-21-hACE2 cells (**Figure 2D**).

### The immune evasion of emerging Omicron subvariants

**Figure 3:**
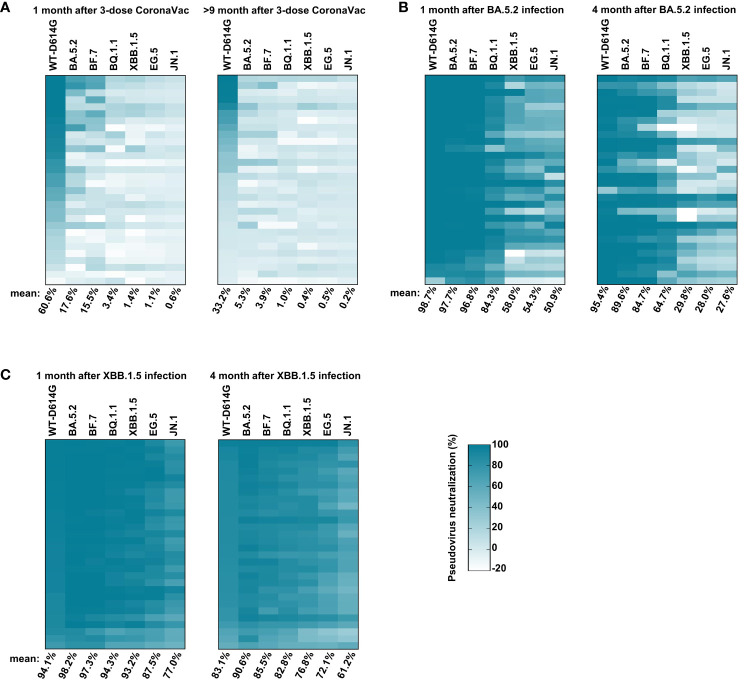
Sera-neutralizing activity against Omicron subvariants. **(A)** The neutralizing activity of vaccinees. The sera samples were collected at 1 month and 9 months after three doses of inactivated vaccine administrations at 25-fold dilution against pseudoviruses bearing S proteins from the indicated variants with WT-D614G as a control. **(B)** The neutralizing activity of vaccinees’ sera at 1 month and 4 months after BA.5.2 infection at 25-fold dilution. **(C)** The neutralizing activity of vaccinees’ sera at 1 month and 4 months after XBB.1.5 reinfection at 25-fold dilution. The color of the small square represents the number of neutralizing activity. The numbers at the bottom of each column donates the mean of the pseudovirus neutralizing activity. Three times of replicates were tested and the average values were taken. “100% pseudovirus neutralization” indicates that 100% of pseudovirus is neutralized.

The emergence of immune evasion SARS-CoV-2 variants repeatedly, especially Omicron subvariants (BA.5.2, BF.7, XBB.1.5, EG.5, etc.), is still a global threat (Wang et al., 2022; Kurhade et al., 2023; Miller et al., 2023). To monitor the immune response against these variants in vaccinees, we first conducted the pseudovirus neutralization assay with sera samples collected at 1 month and 9 months after the third dose of inactivated vaccine administration. One month after the third dose, the serum mean neutralization efficiency against WT-D614G was 60.6%. However, the efficiency reduced to 17.6%, 15.5%, 3.4%, 1.4%, 1.1%, and 0.6% for BA.5.2, BF.7, BQ.1.1, XBB.1.5, EG.5, and JN.1, respectively, when we tested the sera samples at 25-fold dilution, indicating the significant immune evasion of these Omicron subvariants. Nine months after the third dose of inactivated vaccine, the mean neutralization efficiency of the sera against WT-D614G drop to 33.2%, about a half than before. Moreover, the efficiency against Omicron subvariants dropped to near background when tested at 25-fold dilution (**Figure 3A**). By November 2022, it has been more than 9 months since the last vaccination for most people in China. Considering the immune evasion of BA.5.2 and BF.7, the major prevalent subvariants (https://www.chinacdc.cn), as well as the dropped herd immunity established by vaccines, the infection peak of Omicron BA.5.2/BF.7 infection during this period could be explained.

After the infection peak of BA.5.2/BF.7 in China, new variants such as BQ.1.1 and XBB.1.5 were circulating around the world (Callaway, 2023). We conducted the pseudovirus neutralization assay with sera samples collected at 1 month and 4 months after the BA.5.2 infection. One month after BA.5.2 infection, the humoral immune was significantly improved, the serum mean neutralization efficiency was 98.7%, 97.7%, 96.8%, 84.3%, 58.0%, 54.3%, and 50.9% against WT-D614G, BA.5.2, BF.7, BQ.1.1, XBB.1.5, EG.5, and JN.1, respectively. Four months after the BA.5.2 infection, the efficiency against WT-D614G, BA.5.2, BF.7, and BQ.1.1 maintained well, but the efficiency against XBB.1.5 dropped to 29.8% (**Figure 2B**), consistent with the mini-wave of XBB.1.5 in the summer of 2023 in China (**Figure 1**). In addition, the humoral immune against Omicron sublineages was significantly improved by the XBB.1.5 infection. The serum neutralization efficiency against most tested Omicron sublineages kept at relatively high level within 4 months after the reinfection (**Figure 3C**). A potentially unsettling factor is that the neutralization efficiency against JN.1 sublineages dropped to 61.2% at 4 months after the reinfection, indicating the potential infection risk of JN.1 in the spring of 2024 in China.

### The mitigated immune imprint of inactivated vaccine

**Figure 4:**
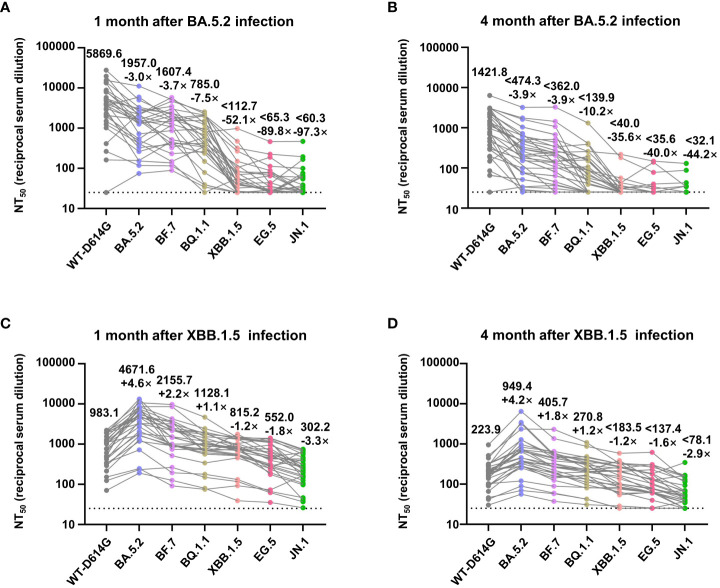
NT_50_ values of convalescents’ sera against Omicron subvariants. **(A, B)** Analysis of NT_50_ values of BA.5.2 convalescents’ sera (1 month and 4 months after the infection) against WT-D614G and indicated Omicron subvariants. **(C, D)** Analysis of NT_50_ values of XBB.1.5 convalescents’ sera (1 month and 4 months after the infection), who had been infected by BA.5.2 previously. The top numbers on top of each column donate the mean of the NT_50_ values, and the bottom numbers on top of each column donate the fold changes of the mean values comparing with the WT-D614G. Dashed lines indicate the limit of detection (NT_50 _= 25).

For further quantitative research on the immune response in vaccinees after breakthrough infection, we assessed the geometric mean 50% neutralization titer (NT_50_) of sera samples collected after infections against the Omicron sublineages. One month after the BA.5.2 infection, the NT_50_ against the WT-D614G, BA.5.2, BF.7, BQ.1.1, XBB.1.5, EG.5, and JN.1 was 5869.6, 1957.0, 1607.4, 785.0, 112.7, 65.3, and 60.3, respectively. Comparing with WT-D614G, the NT_50_ to Omicron subvariants are lower by factors of 3.0–97.3. Four months after the BA.5.2 infection, the NT_50_ decreased by factor of 1.8–5.6, alerting the reinfection risk of XBB.1.5 at that time (**Figures 4A, B**). Notably, we found out that the infection of Omicron BA.5.2 largely boosted the neutralizing antibodies against WT-D614G (**Figures 3A, B**). Meanwhile, the NT_50_ against the WT-D614G was higher than NT_50_ against the BA.5.2 after the BA.5.2 infection (**Figures 4A, B**), indicating the vaccine-induced immune imprinting.

In addition, we also connected another 11 participants who had accepted three doses of inactivated vaccine administrations and then been infected by BF.7 in Huzhou, China. Their immune response at 1 month after BF.7 infection was similar with BA.5.2 convalescents (**Supplementary Figure S1**), which are understandable since there was only one different mutation (R346T) in their Spike (**Figure 2A**). Regretfully, we lost connection with this BF.7 cohort.

Moreover, 1 month after the XBB.1.5 infection, the NT_50_ against the WT-D614G, BA.5.2, BF.7, BQ.1.1, XBB.1.5, EG.5, and JN.1 was 983.1, 4671.6, 2155.7, 1128.1, 815.2, 552.0, and 302.2, respectively. Four months after the XBB.1.5 infection, the NT_50_ decreased by factor of 3.9–5.3 (**Figures 4C, D**). After the infection of XBB.1.5, NT_50_ against the BA.5.2 became the highest rather than NT_50_ against WT-D614G. These results indicated that two rounds of Omicron infections largely mitigated the immune imprint of three doses of inactivated vaccine.

## Discussion

In this study, we built a long-tracked cohort consisting of 30 vaccinees in Wuhan, who were infected by BA.5.2 and XBB.1.5 in 2022 and 2023, respectively (**Figure 1**). By monitoring the humoral immune before and after the infections, we found out that the variants that caused the mini-waves of breakthrough infections were variants with drastic immune evasion, and the decreasing sera neutralization in vaccinees along time aggravated the breakthrough infections (**Figures 3**, **4**). Notably, two rounds of Omicron infections could efficiently mitigate the immune imprint of the original-strain based inactivated vaccine, consisting with the previous reports (Cao et al., 2023; Yisimayi et al., 2024).

The mutations in the Spike, especially in the RBD, lead to the immune evasion abilities of Omicron subvariants. The emerging JN.1 variant, with newly identified N450D and L455S mutations, showed potential immune evasion (**Figures 4C, D**). However, the damage of possible future mini-wave of JN.1 infection will not be as serious as BA.5.2 or BF.7 in China since the herd immunity built by the BA.5.2/BF/7 infection peak. Meanwhile, in spite of the different hACE2 affinity, no significant difference was found in the infectivity of the Omicron subvariants (**Figure 2D**), consisting with the previous reports (Cao et al., 2023; Faraone et al., 2023; Shuai et al., 2022).

Immune imprint is the immune system’s propensity to limit its response to new mutated antigens after responding to the original antigen (Pérez-Alós and Hansen, 2023). Many kinds of vaccines based on the original SARS-CoV-2 have been used around world, and have played important roles in the fight against the COVID-19. However, the immune imprint of these vaccines is reported while the Omicron subvariants emerge. The infection of BA.5.2 and XBB.1.5 mitigated immune imprint in the vaccinees, and improved the Omicron specific immune response, consistent with the studies on other kinds of vaccinees (Yisimayi et al., 2024; Röltgen et al., 2022; Li et al., 2022).

In sum, by monitoring the immune response of a long-tracked cohort, we found out that the immune evasion of emerging Omicron subvariants is largely responsible for the breakthrough infections. Meanwhile, the immune imprint in vaccinees can be largely mitigated by repeated Omicron infections, indicating the necessary of multiple administrations of vaccines and the updating of vaccines rapidly, the vaccines based on XBB variants (Patel et al., 2023; Hansen et al., 2024), for example.

## Methods

### Sample collection

Blood samples from the long-tracked cohort (30 participants) who had three-dose vaccination of inactivated vaccine were collected after 1 month and 9–10 months. The same cohort was infected by Omicron BA.5.2, and all participants recovered within 2 weeks. At 1 month and 4 months after the infection, blood samples from this cohort were collected. The same cohort was then infected by Omicron XBB.1.5, and all individuals recovered within 2 weeks. At 1 month and 4 months after the infection, blood samples from this cohort were collected. Another 11 blood samples were collected at 1 month after the BF.7 infection. After centrifugation, serum from the upper layer were collected and incubated at 56°C for 30 min for heat inactivation, and then stored at −80°C until use. The infecting strain was determined by viral genome sequencing of the throat swab from the participants. The participants were randomly recruited to the cohort according to the criteria of the easy sampling principle. Convalescents who had mental disorders, dementia, difficulty in moving freely, or refused to enroll or were not contactable were excluded from the cohort. The age range for the participants was 19–65 years; 16 participants were male and 14 participants were female.

### Plasmids

The DNA sequences of human codon-optimized S proteins from SARS-COV-2 variants (WT-D614G, Delta, BA.5.2, BF.7, BQ.1.1, XBB.1.5, EG.5, and JN.1) and S protein mutations were commercially synthesized or generated by overlapping PCR-based mutagenesis using pCAGGS-SARS-CoV-2-S-C9 as the template and cloned into pCAGGS vector with C-terminal 18 amino acid (aa) truncation to improve VSV pseudotyping efficiency.

### Cell culture

BHK-21 (ATCC, CCL-10) and Vero E6 cells (ATCC, CRL-1586) were cultured in Dulbecco’s modified Eagle’s medium (DMEM, Gibco) supplemented with 10% fetal bovine serum (FBS, Excell Bio, Suzhou, China), 2.0 mM L-glutamine, 110 mg/L sodium pyruvate, and 4.5 g/L D-glucose. BHK-21-ACE2 (clone 7), which stably expresses human ACE2 was generated based on BHK-21 through lentiviral transduction and maintained with puromycin at 1 μg/mL. I1-Hybridoma (CRL-2700) secreting a monoclonal antibody targeting VSV glycoprotein was cultured in minimum essential medium with Earle’s salts and 2.0 mM L-glutamine (MEM; Gibco) supplemented with 10% FBS. All cells were cultured at 37°C in 5% CO_2_ with regular passage every 2–3 days.

### Expression and purification of Fc-RBD

HEK293F cells were cultured in SMM 293T-I medium (Sino Biological) at 37°C, with 5% CO_2_. Plasmids of Fc-RBD were transfected into HEK293F when the cell density is 1 × 10^6^ cells per mL with EZ Trans (Life-Lilab) transfection reagent. The cell supernatant was collected by centrifugation to remove the cells, and Protein A Resin (GenScript) was used to purify the mAbs. These mAbs were further purified by a Superose 6 10/300 column (GE Life Sciences) and resuspended into phosphate-buffered saline (PBS), and the purity of the recombinant antibodies was analyzed by SDS-PAGE.

### Biolayer interferometry binding assay

The binding affinity of the purified SARS-CoV-2 Fc-RBD protein with His-hACE2 was monitored by BLI using an Octet-Red 96 device (Pall ForteBio). Briefly, 20 μg/ml of Fc-RBD were loaded onto protein A (ProA) biosensors (ForteBio, 18–5010), and then dipped into phosphate-buffered saline with Tween 20 (PBST) buffer to wash out unbound mAb. Then, the biosensors were dipped into PBST buffer containing soluble His-hACE2 protein with concentrations ranging from 0 nM to 300 nM and then dipped into PBST buffer to record dissociation kinetics. PBST buffer was used to define the background. The affinities were calculated by Octet Data Analysis software.

### SARS-CoV-2 pseudovirus production and titration

The pseudovirus packaged with spike proteins from the WT-D614G SARS-CoV-2, and the SARS-CoV-2 variants were produced as we described before (Liu Q. et al., 2022). In brief, Vero-E6 cells were transfected with plasmids expressing different S proteins through Lipofectamine 2000 (Biosharp, Hefei, China). After 24h, the transfected cells were inoculated with VSV-dG-fLuc [1 × 10^6^ 50% tissue culture infectious dose (TCID_50_)/mL] diluted in DMEM for 5h at 37°C and then replenished with growth medium (DMEM with 10% FBS) containing anti-VSV-G monoclonal antibody (I1-hybridoma, cultured supernatant, 1:20). Twenty-four hours later, the SARS-CoV-2 pseudovirus-containing supernatant was harvested and clarified at 3,000 rpm for 10 min, aliquoted, and frozen at −80°C for storage. The TCID_50_ of the pseudovirus was determined using a serial dilution-based infection assay on BHK-21-hACE2 cells and calculated according to the Reed-Muench method.

### SARS-CoV-2 pseudovirus neutralization assay

The SARS-CoV-2 pseudoviruses (1 × 10^5^) TCID_50_/well) were incubated with serial-diluted sera at room temperature for 30 min in 96-well white flat-bottom culture plates and then mixed with trypsinized BHK-21-hACE2 cells at a density of 2 × 10^4^/well. After 16h, the medium of the infected cells was removed, and the cells were lysed with 1× Bright-Glo Luciferase Assay reagent (Promega) for chemiluminescence detection using a SpectraMax iD3 multi-well luminometer (molecular devices). The 50% neutralization dilution titer (NT_50_) was calculated by GraphPad Prism 7 software with nonlinear regression curve fitting (normalized response, variable slope).

## Data availability statement

The raw data supporting the conclusions of this article will be made available by the authors, without undue reservation.

## Ethics statement

The studies involving humans were approved by The Institutional Review Boards of Hubei Provincial Centre for Disease Control (2021-012-01) and the Life Medical Ethics Committee at Wuhan University (WHU-LFMD-IRB2023002). Informed consent was obtained from all human research participants. The studies were conducted in accordance with the local legislation and institutional requirements. The participants provided their written informed consent to participate in this study.

## Author contributions

QL: Conceptualization, Writing – review & editing, Formal analysis, Funding acquisition, Investigation, Methodology, Software, Validation, Visualization, Writing – original draft. MJ: Project administration, Resources, Validation, Writing – review & editing, Conceptualization, Investigation. FM: Conceptualization, Funding acquisition, Investigation, Project administration, Resources, Validation, Writing – review & editing. HF: Conceptualization, Data curation, Investigation, Methodology, Resources, Visualization, Writing – review & editing. MG: Data curation, Investigation, Resources, Software, Validation, Visualization, Writing – original draft. YZ: Data curation, Formal analysis, Investigation, Methodology, Software, Validation, Writing – review & editing. SQ: Data curation, Formal analysis, Investigation, Methodology, Resources, Software, Validation, Writing – review & editing. XT: Data curation, Formal analysis, Investigation, Methodology, Software, Writing – review & editing. LJ: Conceptualization, Data curation, Resources, Writing – review & editing. ZZ: Data curation, Investigation, Software, Validation, Writing – review & editing. GC: Investigation, Validation, Visualization, Writing – review & editing. HY: Investigation, Supervision, Writing – review & editing. YC: Investigation, Project administration, Supervision, Validation, Writing – review & editing, Resources. KL: Investigation, Project administration, Resources, Supervision, Writing – review & editing, Conceptualization, Funding acquisition. QG: Investigation, Project administration, Resources, Supervision, Writing – review & editing, Validation. KC: Conceptualization, Investigation, Project administration, Resources, Supervision, Writing – review & editing, Data curation. LZ: Conceptualization, Funding acquisition, Investigation, Project administration, Resources, Supervision, Writing – review & editing.

## Supplementary material

Supplementary Figure 1 Analysis of NT_50_ values of BF.7 convalescents’ sera (1 month after the infection) against WT-D614G and indicated Omicron subvariants. The top numbers on top of each column donate the mean of the NT_50_ values, and the bottom numbers on top of each column donate the fold changes of the mean values comparing with the WT-D614G. Dashed lines indicate the limit of detection (NT_50 _= 25).
