# Supplementary figures and images for: A long-term cohort study: the immune evasion and decreasing neutralization dominated the SARS-CoV-2 breakthrough infection
Source: Front Cell Infect Microbiol. 2024 Mar 20;14:1381877. doi: 10.3389/fcimb.2024.1381877 (PMC10987703; doi:10.3389/fcimb.2024.1381877)

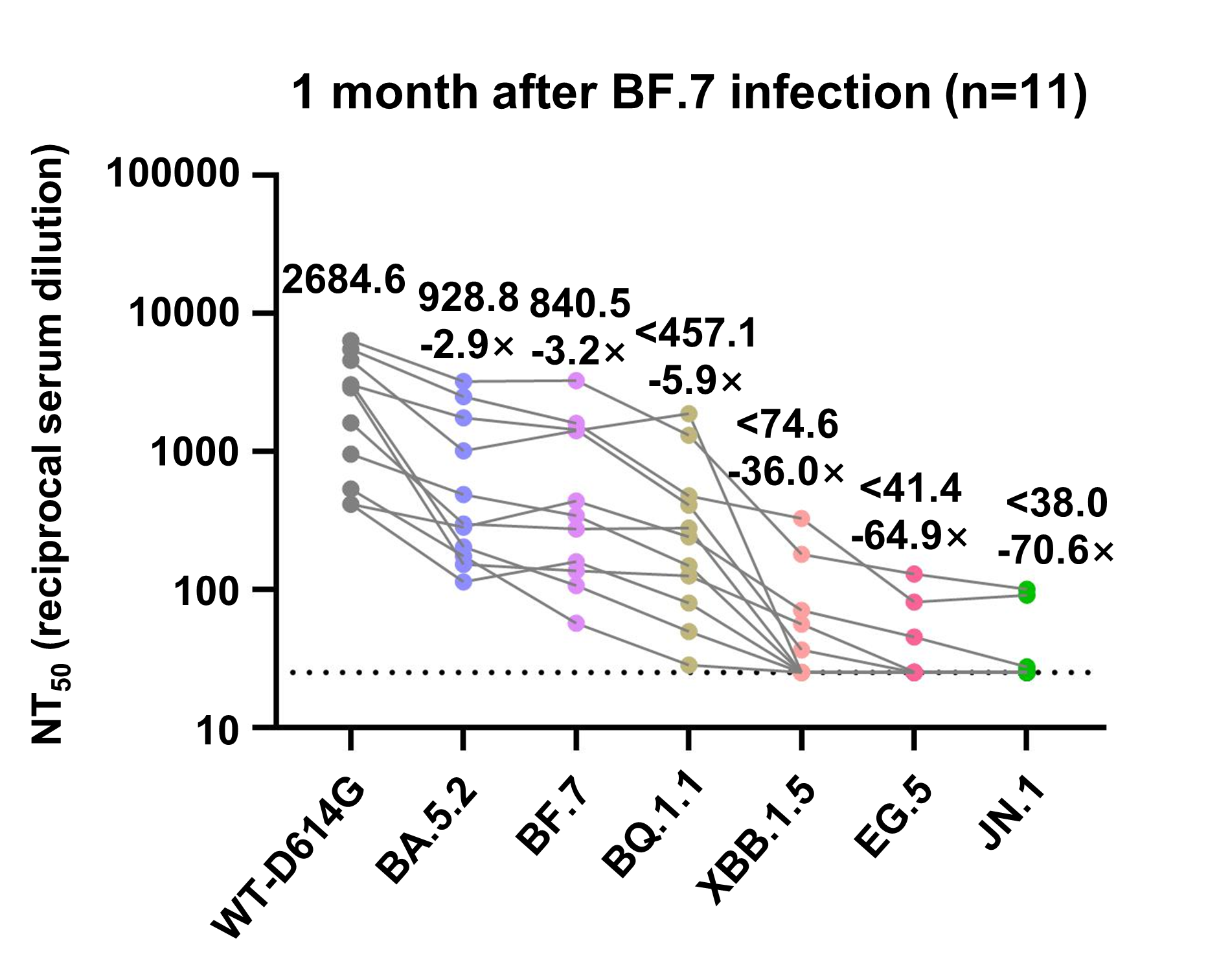

Supplement: Supplementary Figure 1 — Analysis of NT50 values of BF.7 convalescents’ sera (1 month after the infection) against WT-D614G and indicated Omicron subvariants. The top numbers on top of each column donate the mean of the NT50 values, and the bottom numbers on top of each column donate the fold changes of the mean values comparing with the WT-D614G. Dashed lines indicate the limit of detection (NT50 = 25). [file Image_1.tif]
